# Repellency of Plant Extracts against the Legume Flower Thrips *Megalurothrips sjostedti* (Thysanoptera: Thripidae)

**DOI:** 10.3390/insects6030608

**Published:** 2015-06-26

**Authors:** Andnet Abtew, Sevgan Subramanian, Xavier Cheseto, Serge Kreiter, Giovanna Tropea Garzia, Thibaud Martin

**Affiliations:** 1 International Centre of Insect Physiology and Ecology (*icipe*), Nairobi 30772, Kenya; E-Mails: ssubramania@icipe.org (S.S.); xcheseto@icipe.org (X.C.); thibaud.martin@cirad.fr (T.M.); 2 Montpellier SupAgro, UMR CBGP CIRAD/INRA/IRD/SupAgro, Campus International de Baillarguet, CS 30016, Montferrier-sur-Lez 34988, France; E-Mail: serge.kreiter@supagro.fr; 3 Dipartimento di Agricoltura, Alimentazione e Ambiente (Di3A), University of Catania, Via Santa Sofia 100, Catania 95123, Italy; E-Mail: gtgarzia@unict.it; 4 Centre de coopération internationale en recherche agronomique pour le dévelopement (CIRAD), UPR HORTSYS, Montpellier F-34398, France

**Keywords:** plant extracts, repellency, *Megalurothrips sjostedti*, Olfactometer, *Piper nigrum*

## Abstract

*Megalurothrips sjostedti* Trybom is an important pest of cowpea (*Vigna unguiculata* L.) in Africa. To propose an alternative to chemical control, the repellency of 24 plant extracts was evaluated against adult female thrips of *M. sjostedti* in the laboratory. Plant extracts in ethanol were separately applied on a filter paper disk in a still air visual cue olfactometer. The results showed highly significant differences in repellency among extract type, concentration and their interactions. We classified the level of repellency into four categories as strong, good, moderate and weak or non- repellent based on hierarchical ascendant classification. We identified *Piper nigrum*, *Cinnamomum zeylanicum*, *Cinnamomum cassia* as strong repellents. Five extracts were classified as good, eight as moderate and the remaining eight extracts were weak or non-repellent. Repellency of the extracts increased with the concentration suggesting that the behavioral response of *M. sjostedti* was dose-dependent. Mono- and sesquiterpene hydrocarbon compounds from seven highly repellent extracts were identified by gas chromatography-mass spectrometry (GC/MS). The use of repellent extracts could be useful in developing integrated pest management strategies for thrips on legume crops. In this regard, the specific modes of action of the identified compounds need to be investigated to incorporate them into the existing crop protection strategies.

## 1. Introduction

The Legume Flower Thrips (LFT), *Megalurothrips sjostedti* Trybom (Thysanoptera: Thripidae), is one of the most serious insect pests of leguminous plants including cowpea in tropical Africa [1,2,3,4]. Thrips occur on legumes in every growing season, and their direct feeding causes destruction of buds and flowers as well as malformations of pods [5]. Yield losses ranging from 20% to 100% have been reported on cowpea (*Vigna unguiculata* L.) from different areas of Africa where modern pest control measures are absent [1,4].

Currently, the control of *M. sjostedti* in Sub-Saharan African countries relies heavily on synthetic insecticide application [6]. The indiscriminate use of these chemicals has given rise to problems such as resistance of the legume pests to insecticides [7,8], accumulation of toxic residues in food, health risks to the consumer and livestock and environmental contamination [7,9,10]. As a result, there is an urgent need to develop alternatives, which are safe, effective, biodegradable and highly selective. Pesticides from plant-based extracts have been suggested as a better alternative to synthetic insecticides [11].

Plant extracts contain many secondary metabolites that act as repellents, feeding deterrents and toxins, which have a role in defense against herbivores, pests and pathogens [12]. These secondary metabolites are released in the form of plant volatiles. Plant extracts are a complex mixture of general leaf volatiles, found in most plant species with more specific components that are shared by some plant species groups [13]. Essential oils generally consist of several constituents produced as secondary plant metabolites, the majority of which are terpene hydrocarbons, polyphenolic compounds and alkaloids [14]. Essential oils from different plant species are an important source of repellents. These odors have been extensively tested for safety and toxicity and have shown no deleterious effects on beneficial insects and are therefore considered to be one of the new means of crop protection [15,16]. Hence the use of essential oils for pest management is becoming popular, and many new applications are under investigation [13,17,18,19,20].

The release of repellent volatiles into the air by associated plants may disrupt the olfactory orientation of insects such as thrips [21]. Integrating naturally occurring repellent volatiles that defend plants by irritating insects and thereby leading to them spending a shorter time period in a treated area may help reduce insect damage to crops [22]. However, profound knowledge about the behavioral response of the target pest to the specific compound is a precondition for successful utilization of biologically active secondary plant compounds in crop protection strategies [18]. Although several repellent plant extracts and essential oils have been identified against onion thrips and western flower thrips [13,20], there is limited information on the use of repellents against *M. sjostedti*.

Thus, the aim of our study was to investigate potential repellent plant extracts, which could be integrated for the management of thrips in grain legumes and to characterize constituent volatiles released from highly repellent extracts. Integrating repellents that modify pest behavior in conventional legume flower thrips management strategies might improve efficacy in the management of thrips in the small holder farmers in Sub-Saharan African countries.

## 2. Experimental Section

### 2.1. Insect Culture and Rearing

The initial culture of adult *M. sjostedti* was field-collected from pigeon pea, Cajanus cajan (L.) Millsp. in Matuu, Yatta district, Kenya (1°16′N; 37°53′E; 1246 m a.s.l.). The thrips were subsequently reared in ventilated plastic jars provided with French bean (*Phaseolus vulgaris* L.) pods as described by Ekesi *et al.* [3]. The insect cultures were maintained in a laboratory at *icipe*, Nairobi, Kenya at 26 ± 2 °C; 60% ± 5% RH and 12L:12D photoperiod. Bean pods containing fresh thrips eggs were transferred to new jars to obtain adult thrips of known age. Adult female thrips (four to five days old) were used in the bioassays. To avoid behavioral bias prior to experimentation, adult thrips were conditioned for half a day without beans in empty jars.

### 2.2. Olfactometer Bioassay

**Figure 1:**
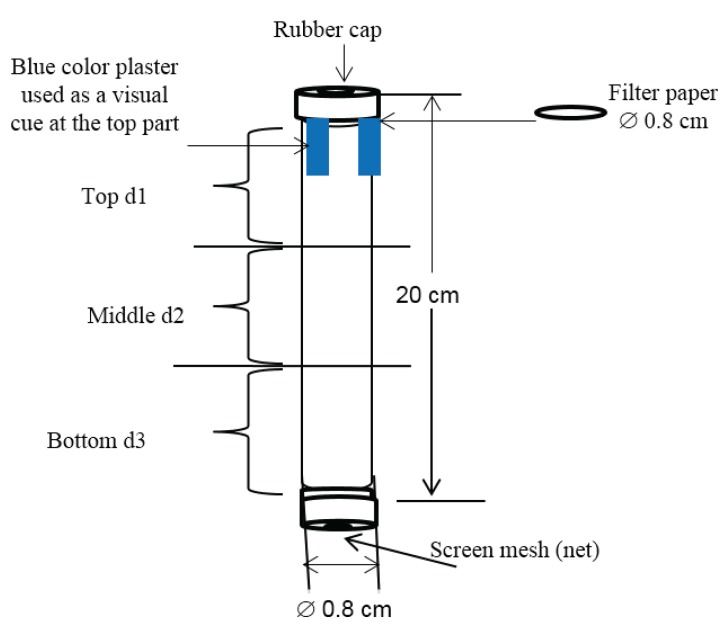
Schematic representation of simple tube still-air visual and odor cue olfactometer.

To compare repellent effects of plant extracts against *M. sjostedti*, we adapted a simple tube still-air visual and odor cue olfactometer used by Deletre *et al.* [23,24] as detailed in Figure 1. The olfactometer measured 20 cm in length and 0.8 cm internal diameter. The tube was divided into three equally partitioned sections: top, middle and bottom. The top section of the tube was covered with a blue colored 3M Vinyl electrical tape (Taiwan Scotch™) with a size of 2.4 cm × 4.5 cm as a visual cue (Figure 1). The bioassay was carried out in a completely randomized design (CRD) experimental set up at room temperature (26 ± 2 °C) inside a fume hood (Telstar BIO II) illuminated with fluorescent light. All seven olfactometer tubes were placed vertically on a test tube rack, parallel to each other inside the hood. The still-air visual cue olfactometer used in the study provided a combination of visual (blue color) and olfactory cues (plant extracts at different concentrations) in the test for repellency. A combination of visual and olfactory cues is involved in responses of thrips to host cues [25,26] including *M. sjostedti* [27].

Plant extracts tested in repellency bioassays were sourced from different suppliers as listed in Table 1. Aliquots (3 μL) of plant extracts at three selected concentrations prepared in absolute ethanol (0.01%, 0.1% and 1%) were applied on a piece of filter paper (Ø 0.8 cm) using a pipette (Finnpipette^®^ 2–20 μL Thermo Labsystems, Vantaa, Finland ) and placed at the top end part of the tube. Ten female *M. sjostedti* thrips were released at the bottom end of each tube using a small aspirator. The top end of the tube was closed with a rubber cap to prevent the odor from escaping whereas the other end was closed using a silk micro-screen fine mesh to avoid escaping of the insects and ensure ventilation. The number of insects in the three parts was counted 5 and 10 min after introduction. Seven replicates were run for each treatment, which were comprised of different concentrations of plant extracts. Absolute ethanol (Scharlad S.L., Spain), which was used as solvent for the plant extracts was used as a control in each experiment. The treatments were only different doses of odor cues, and the visual cue was the same for all treatments and controls. Since the experiments were repeated at different times, the control solution was run with each experiment. To avoid a dose effect, a new cohort of *M. sjostedti* was assayed for each concentration taken from the batch of starved and conditioned adult thrips.

### 2.3. Data Collection

To determine repellency, the number of thrips was recorded for the three sections of the tube 5 and 10 min after odor introduction. Based on the observations, mean repellency was estimated as described below:

(1) S=((mdT*t)+(mdM*m)+(mdB*b))/n

where *S* is the mean repellency per thrips for each extract or essential oil, mdT is the mean distance for the top section = 3.3 cm, mdM is the mean distance of thrips movement for the middle section = 10.0 cm, mdB is the mean distance of thrips movement for the bottom section = 16.5 cm, t is the mean number of insect count at the top, m is the number of insect count at the middle, b is the number of insect count at the bottom and n is the total number of insects per tube (n = t + m + b).

### 2.4. GC-MS Analysis

Aliquots (1 μL) of samples from the top seven repellent plant extracts from the olfactometer assay, prepared at a concentration of 1000 μg were analyzed with gas chromatography (GC) (Agilent 7890A) capillary column (HP-5, 30 m, 0.25 mm, i.d. 0.25 μm) directly coupled to a mass spectrometer (MS) (Agilent, Palo Alto, CA, USA) to identify the component compounds of the extracts. Ionization was performed by electron impact (70 eV, 230 °C). The oven temperature was maintained at 35 °C for 3 min and then programmed at 10 °C to 285 °C·min^−1^. The carrier gas was helium at a flow rate of 1.25 mL·min^−1^. Compounds were identified by comparison of retention indices and mass spectra with those of authentic standards. In the absence of corresponding reference compounds, structures were proposed on the basis of MS fragmentation pattern combined with reference spectra in the database (NIST 05, NIST 08, Adams and chemical).

**Table 1:** Names and sources of the plant extracts used for the repellency experiment.

No	Common Name	Scientific Name	Family	Extract Type, Plant Part Used	Supplier, Country
1	African blue basil	*Ocimum kilimandscharicum*	*Lamiaceae*	Essential oil, leaf	*icipe*—Bioprospecting unit, Kenya
2	Black pepper	*Piper nigrum*	*Piperaceae*	Essential oil, seed	IBMM ^1^, France
3	Ceylon cinnamomum	*Cinnamomum zeylanicum*	*Lauraceae*	Essential oil, inner bark	Nactis, France
4	Chinese cinnamomum	*Cinnamomum cassia*	*Lauraceae*	Essential oil, bark	Huiles & Sens, France
5	Citronella	*Cymbopogon nardus*	*Poaceae*	Essential oil, leaf	Burgess & Finch, South Africa
6	Conyza	*Conyza newii*	*Asteraceae*	Essential oil, leaf	*icipe*—Bioprospecting unit, Kenya
7	Coriander	*Coriandrum sativum*	*Umbelliferae*	Essential oil, seed	Fabster, France
8	Dill	*Anethum graveolens*	*Apiaceae*	Essential oil, seed	IBMM, France
9	Eucalyptus	*Eucalyptus globulus*	*Myrtaceae*	Essential oil, leaf	Huiles & Sens, France
10	Geranium	*Pelargonium graveolens*	*Geraniaceae*	Essential oil, leaf	IBMM, France
11	Ginger	*Zingiber officinale*	*Zingiberaceae*	Essential oil, root	Burgess & Finch, South Africa
12	Lemon	*Citrus limon*	*Rutaceae*	Essential oil, fruit	Capua, Italy
13	Lemon grass	*Cymbopogon citratus*	*Poaceae*	Essential oil, leaf	Burgess & finch, South Africa
14	Lemon savory	Satureja biflora	*Lamiaceae*	Essential oil, leaf	*icipe*—Bioprospecting unit, Kenya
15	Marjoram	*Origanum majorana*	*Labiatae*	Essential oil, leaf	Burgess & Finch, South Africa
16	May chang	*Litsea cubeba*	*Lauraceae*	Essential oil, fruit	IBMM, France
17	Myrrha	*Commiphora myrrha*	*Burseraceae*	Essential oil, oleoresin-gum	Burgess & Finch, South Africa
18	Neem	*Melia azadirachta*	*Meliaceae*	Vegetable oil, seed	Huiles & Sens, France
19	Pennyroyal	*Mentha pulegium*	*Lamiaceae*	Essential oil, leaf	IBMM, France
20	Rosemary	*Rosmalinus officinalis*	*Lamiaceae*	Organic floral water , leaf	Huiles & Sens, France
21	Savory	*Satureja abyssinica*	*Lamiaceae*	Essential oil, leaf	*icipe*—Bioprospecting unit, Kenya
22	Solidage	*Solidago canadensis*	*Asteraceae*	Essential oil, flower	Huiles & Sens, France
23	Thyme (wild)	*Thymus satureioides*	*Lamiaceae*	Essential oil, flower	Huiles & Sens, France
24	Thyme (common)	*Thymus vulgaris*	*Lamiaceae*	Essential oil, leaf	Burgess & Finch, South Africa

^1^ IBMM—Institut des Biomole’cules Max Mousseron, Montpellier, France.

### 2.5. Quantification of Terpenes

GC-MS in full scan mode was used to detect terpenes in the oil and plant extracts. Serial dilutions of Limonene and Caryophyllene oxide (1–100 pg/µL) were analyzed by GC-MS in scan mode to generate linear calibration curves (peak area *vs.* concentration) with the following equations; Limonene (y = 8E + 06x *R*^2^ = 0.9979) for monoterpenes and Caryophyllene oxide (y = 4E + 06x − 2E + 07; *R*^2^ = 0.9584) for sesquiterpenes. The two compounds were randomly selected based on their column chemistries in relation to the target class of compounds. Relative percentages of each compound in compositions of essential oils were calculated based on the corresponding areas of the identified compounds.

### 2.6. Data Analysis

The count data of thrips observed at the three sections of the olfactometer were converted to continuous data of mean repellency as detailed earlier. Data analysis was carried out using Analysis of Variance (ANOVA). Since the response to the control did not vary significantly among the different experiments, a mean control value was used in the statistical analysis. Where the ANOVA showed significant differences of the interactions between concentration and extract, pairwise comparison of the concentrations of each extract with the control was performed using the Student Newman Keuls test. The differences in thrips responses among different extracts for each dose were also tested with the control and a comparison of means was implemented using the Student Newman Keuls test.

The pooled interaction effect of plant extracts and concentrations was further explored using principal component analysis (PCA). Then, based on the similarity of their repellent effect, a hierarchical ascendant classification (HAC) on ward’s algorithm was used to group the plant extracts using PCA-axes coordinates. This process yielded a binary segmentation tree (dendrogram), reflecting the hierarchy of similarities between *M. sjostedti* responses to plant extracts. The optimal number of classes in the tree was determined by the decrease of the interclass variance (branch height-Supplement Figure 1). The analyses were implemented using R version 3.11 (R core team 2014) [28].

## 3. Results

### 3.1. Olfactometer Bioassays

Among the three observation sections of the olfactometer, 52% of the thrips were observed in the top of the olfactometer (where the visual and olfactory cues were presented), while 34% and 13% of the thrips were observed at the bottom and the middle of olfactometer, respectively, when considered irrespective of doses, extracts and time of exposure (F_2, 3063_ = 1840, *p* < 0.001). Analysis of the mean repellency indicated that the effect of the interaction between extract type, concentration used and time on mean thrips repellence was not significant (F_69, 1152_ = 0.56, *p* = 1). The two ways interaction effects between extract and time and concentration and time were not significant either (F_23, 1152_ = 0.88, *p* = 0.63; F_3, 1152_ = 1.94, *p* = 0.121, respectively). However, the interaction between extract and concentration was highly significant (F_69, 1152_ = 3.43, *p* < 0.001). The main effects of concentration and extracts were highly significant; F_3,1152_ = 143.3, *p* < 0.001 and F_23,1152_ = 8.65, *p* < 0.001, respectively) while the main effect of time (5 min and 10 min) after odor introduction was not significant (F_1,1152_ = 0.43, *p* = 0.51).There was no significant difference in mean repellency between the two ways interaction effects, extract types and time (F_23, 1152_ = 0.88, *p* = 0.62), and concentration and time (F_3, 1152_ = 1.94, *p* = 0.12) and between the times of 5 and 10 min after odor introduction (F_1, 1152_ = 0.43, *p* = 0.51).

In general, the repellency of extracts increased as the concentration of plant extract/essential oils increased from 0.01% to 1%. At a lower concentration (0.01%), the oils of *P. nigrum*, *C. zeylanicum*, *C. cassia*, *C. citratus* were significantly different in repelling *M. sjostedti*, and the repellency ranged from 9.2 to 10.3 cm distance from the odor source (F_24,326_ = 5.33, *p* < 0.001). At a 0.1% concentration, repellency of *P. nigrum*, *C. zeylanicum*, *C. cassia*, *E. globulus*, *C. myrrha Z. officinale*, *T. vulgaris* and *P. graveolens* were significantly different from the control, and repellency ranged from 9.3 to 11.3 cm (F_24, 326_ = 5.15, *p* < 0.001). At a 1% concentration, *P. nigrum*, *C. zeylanicum*, *C. cassia*, *E. globulus*, *C. myrrha*, *T. vulgaris*, *M. pulegium*, *C. citrates*, *C. nardus*, *S. biflora*, *M. azadirachta*, *A. graveolens*, *L. cubeba*, *C. sativum*, *O. majorana*, *S. abyssinica* and *P. graveolens* were significantly different from the control, and repellency ranged from 9.1 to 11.8 cm (F_24, 326_ = 5.99, *p* < 0.001). At all three doses, *T. satureioides*, *O. kilimandscharicum*, *S. canadensis*, *C. newii*, *C. limon* and *R. officinalis* were not significantly different from the control for repellency of *M. sjostedti* (Table 2). 

**Figure 2:**
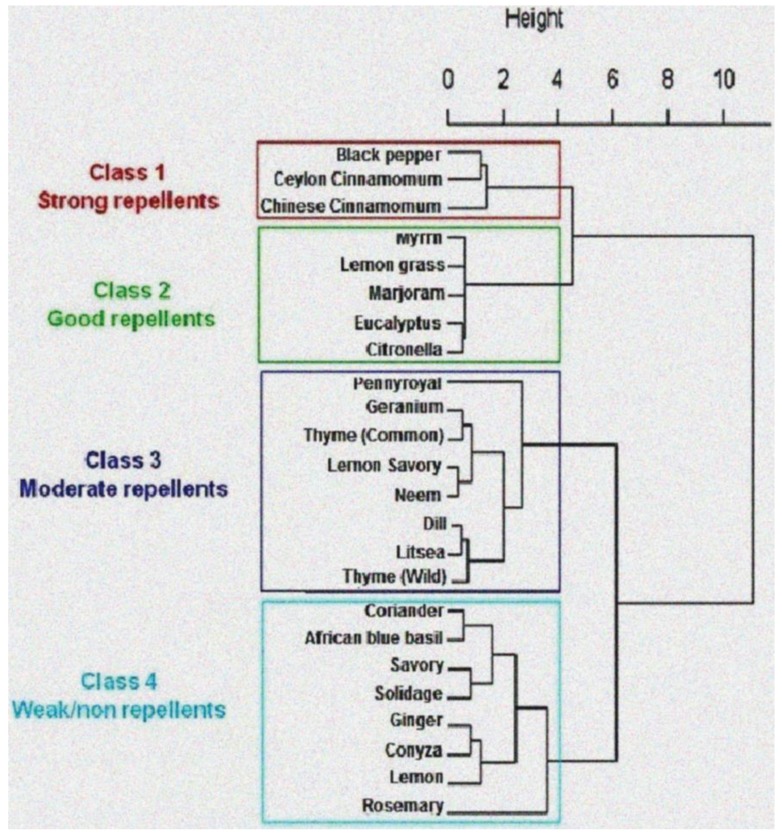
Dendrogram representing response of female *M. sjostedti* to the pooled interaction effect of extracts and concentrations.

Based on the Hierarchical Ascendant Classification (HAC) analysis of repellence effects, the different plant extracts were categorized into to four classes: strong (Class 1), good (Class 2), moderate (Class 3) and weak or non repellent (Class 4). *Piper nigrum*, *C. zeylanicum* and *C. cassia* extracts were classified as strong repellents (Class 1), while *C. myrrha*, *C. citratus*, *O. majorana*, *E. globulus* and *C. nardus* were found to be good repellents (Class 2). Eight extracts were moderately repellent (Class 3); these were: *M. pulegium*, *P. graveolens*, *T. vulgaris*, *S. biflora*, *M. azadirachta*, *A. graveolens*, *L. cubeba*, and *T. satureioides*. As compared to the other extracts, the remaining eight extracts were weak or non repellent (Class 4). These were: *C. sativum*, *O. kilimandscharicum*, *S. abyssinica*, *S. canadensis*, *Z. officinale*, *C. newii*, *C. limon* and *R. officinalis* (Figure 2).

**Table 2:** Response of female *Megalurothrips sjostedti* to the repellent effect of 24 plant extracts at three concentrations (0.01%, 0.1% and 1%) of extract solution and control on filter paper.

S.N	Extract	Concentration (%)				SE of Mean
		0 (control)	0.01	0. 1	1	
		6.9				
1	Black pepper		10.3 *	11.3 *	10.1 *	±0.59
2	Ceylon Cinnamomum		9.4 *	9.7 *	10.5 *	±0.35
3	Chinese Cinnamomum		9.5 *	9.3 *	11.8 *	±0.41
4	Myrrh		8.7	9.9 *	9.9 *	±0.38
5	Lemongrass		9.2 *	9.0	9.8 *	±0.41
6	Marjoram		8.9	8.9	9.2 *	±0.43 ^NS^
7	Eucalyptus		8.6	9.7 *	9.5 *	±0.46
8	Citronella		8.4	9.2	9.8 *	±0.43
9	Pennyroyal		6.8	9.0	11.1 *	±0.35
10	Geranium		7.6	9.3 *	9.8 *	±0.36
11	Thyme (Common)		7.9	10.1 *	9.4 *	±0.49
12	Lemon savory		8.1	8.9	9.7 *	±0.42
13	Neem		7.7	8.8	9.6 *	±0.28
14	Dill		7.3	9.0	9.4 *	±0.38
15	Litsea		8.5	7.2	9.3 *	±0.39
16	Thyme (wild)		6.9	8.9	8.9	±0.43
17	Coriander		8.5	8.3	9.3 *	±0.36
18	African blue basil		8.3	9.1	6.6	±0.36
19	Savory		7.7	7.5	9.1 *	±0.38
20	Solidago		7.7	7.5	8.3	±0.34 ^NS^
21	Ginger		7.6	9.3 *	8.4	±0.37
22	Conyza		7.4	8.6	8.5	±0.39 ^NS^
23	Lemon		8.1	8.4	7.6	±0.45 ^NS^
24	Rosemary		6.6	6.6	7.7	±0.38 ^NS^
	**SE of mean**		±0.43	±0.46	±0.48	±0.59

Within concentrations of 0.01%, 0.1% and 1%, asterisks “*” indicate a significant difference in thrips repellence of extracts from control. Within a row ^NS^ indicates no significant difference in thrips repellence across concentrations for the extract, while all other extracts differed significantly for thrips repellence across concentrations (Student Newman Keul test, *p* = 0.05).

### 3.2. Chemical Characterization

Identification of the chemical constituents from seven highly repellent extracts showed that mono- and sesquiterpenes were the most abundant (Table 3). Fifteen compounds were identified from *P. nigrum* extracts. The major compounds were β-caryophyllene (45.9%), caryophyllene oxide (12.9%) and α-copaene (12.3%). Cinnamaldehyde (79.6%), *trans*-cadina-1(6) and 4-diene (13.24%) were the most abundant compounds identified from *C. zeylanicum* extract. On the other hand, cinnamaldehyde (76.7%) and (E)-ortho-methoxy cinnamaldehyde (16.1%) were the most abundant compounds identified from the extracts of *C. cassia*. Six compounds were identified from *C. myrrha* extracts with curzerene (75.3%) and β-elemene (13.5%) being the two major constituents. Five compounds were identified from extracts of *E. globules* of which 1,8-cineole (93.4%) was the major compound. The major compounds identified from *C. citrates* extracts were geranial (38.3%) and neral (33.7%). Seventeen compounds were identified from *O. marjorana* with trans-sabinene hydrate (16%), terpinene-4-ol (17.9%) and γ-terpinene (11%) being the major component compounds. The most frequently detected component compounds were limonene (detected from four different extracts), α-copaene and β-caryophyllene (detected from three different extracts), α-humulene, camphene, δ-3-carene, cinnamaldehyde, β-selinene, α-muurolene and caryophyllene oxide (detected from two different extracts) (Table 3). Major compounds of the other 17 extracts are presented in the (Supplement Table S1) from different sources.

## 4. Disscussion

The still-air visual and odor cue olfactometer used in the present study provided the opportunity to test the repellency response of legume flower thrips (LFT) to a combination of visual and olfactory cues, which is in line with the host response behavior of thrips [25,26]. However, we observed that a significantly higher proportion of LFT preferred to move to the top section of the olfactometer where the visual cue (blue color) was presented. This could be due to fact that flower/anthophilous thrips are highly attracted to flower color [26,29,30]. Recently LFT was reported to be highly attracted to a combination of flower volatiles and blue color, followed by blue color alone compared to yellow color with or without flower volatiles and flower volatiles alone [27]. The role of negative geotaxactic behavior as reported in thrips species such as western flower thrips [31] and thrips species infesting cereals [32] cannot be discounted but needs to be further tested for LFT. Following the top section, thrips preferred to remain in the bottom section of the olfactometer, which is due to the repellency of the extracts tested. Such an interaction of color and volatile-based cues in eliciting a response of thrips needs to be considered when further refining the still-air visual cue olfactometer, which was used for the first time in this study.

Based on the repellency response of LFT to 24 plant extracts, most of the extracts were repellent to *M. sjostedti*. Black pepper, Ceylon cinnamomum and Chinese cinnamomum were identified as strong repellents, while myrrh, lemon grass, marjoram, eucalyptus and citronella were classified as good repellents. To our knowledge, this is the first study looking at repellency of essential oils against *M. sjostedti*. Generally, the repellency of most extracts varied with concentration and indicated a dose dependent behavioral response of *M. sjostedti.* Our result corroborate previous observations on the repellent effect of essential oils and plant volatiles on other thrips species such as the western flower thrips (WFT), *Frankliniella occidentalis* (Pergande) [20,33,34] and the onion thrips, *Thrips tabaci* (Lindeman) [13,35].

**Table 3:** Compounds of the top seven plant extracts identified using gas chromatography—mass spectrometry (GC-MS).

No	Name	Retention Time (min)	Types of Essential Oils and Relative Percentage (%)						
			*P. nigrum*	*C. zeylanicum*	*C. cassia*	*C. myrrha*	*C. citratus*	*O. marjorana*	*E. globulus*
1 ^◊^	α-Phellandrene	9.69	–	–	–	–	–	1.34	–
2 *	α-Pinene	9.83	–	–	–	–	–	0.74	1.71
3 *	Camphene	10.21	–	–	–	–	1.44	–	0.02
4 ^◊^	Sabinene	10.68	0.08	–	–	–	–	8.95	–
5 *	Myrcene	11.04	–	–	–	–	1.15	1.79	–
6 *	δ-3-Carene	11.40	0.37	–	–	0.94	–	–	–
7 *	δ-2-Carene	11.53	–	–	–	0.57	–	8.45	–
8 *	Limonene	11.75	3.43	–	–	2.10	0.72	3.09	3.09
9 *	β-Pinene	11.76	–	–	–	–	–	4.38	–
10 *	1,8-Cineole	11.80	–	–	–	–	–	–	93.43
11 *	(Z)-Ocimene	11.91	–	–	–	–	0.40	–	–
12 ^◊^	γ-Terpinene	12.31	–	–	–	–	–	12.7	1.75
13 ^◊^	Sabinene hydrate=cis->	12.47	–	–	–	–	–	4.26	–
14 ^◊^	Terpinolene	12.83	–	–	–	–	–	3.22	–
15 *	Linalool	13.01	–	–	–	–	2.06	–	–
16 ^◊^	Sabinene hydrate=trans->	13.03	–	–	–	–	–	18.59	–
17 ^◊^	Menth-2-en-1-ol=cis-para->	13.39	–	–	–	–	–	1.58	–
18 ^◊^	(E)-Isocitral	14.33	–	–	–	–	2.78	–	–
19 *	Terpinen-4-ol	14.33	–	–	–	–	–	20.79	–
20 *	α-Terpineol	14.51	–	–	–	–	0.55	4.87	–
21 ^◊^	Neral	15.29	–	–	–	–	33.66	–	–
22 *	Linalool acetate	15.41	–	–	–	–	–	2.29	–
23 *	Geraniol	15.43	–	–	–	–	5.98	–	–
24 ^◊^	Geranial	15.72	–	–	–	–	38.32	–	–
25 *	(E)-Cinnamaldehyde	15.79	–	79.6	76.7	–	–	–	–
26 ^◊^	δ-Elemene	16.64	7.0	–	–	–	–	–	–
27 ^◊^	α-Cubebene	16.82	1.00	–	–	–	–	–	–
28 ^◊^	Geranyl propanoate	17.18	–	–	–	–	6.4	–	–
29 ^◊^	α-Copaene	17.20	12.3	1.49	0.7	–	–	–	–
30 ^◊^	β-Elemene	17.40	2.5	–	–	13.52	1.16	–	–
31 *	β-Caryophyllene	17.80	45.9	–	–	–	3.78	3.07	–
32 ^◊^	Sesquithujene	17.94	1.65	–	–	–	–	–	–
33 ^◊^	(E)-Cinnamyl acetate	18.05	–	–	0.74	–	–	–	–
34 *	α-Humulene	18.25	3.05	–	–	–	0.95	–	–
35 *	allo- Aromadendrene	18.34	–	–	0.55	–	–	–	–
36 ^◊^	γ-Muurolene	18.50	–	2.80	–	–	–	–	–
37 ^◊^	β-Selinene	18.68	1.73	–	–	7.60	–	–	–
38 ^◊^	Curzerene	18.74	–	–	–	75.27	–	–	–
39 ^◊^	α-Muurolene	18.79	1.76	0.54	–	–	–	–	–
40 ^◊^	Bicyclogermacrene	19.79	–	–	–	–	–	1.10	–
41 ^◊^	γ- Cadinene	18.99	–	0.7	–	–	–	–	–
42 ^◊^	δ- Cadinene	19.08	3.35	–	–	–	–	–	–
43 ^◊^	*trans*- Cadina-1(6),4-diene	19.08	–	13.24	–	–	–	–	–
44 ^◊^	(E)-Methoxy cinnamaldehyde	19.21	–	–	16.1	–	–	–	–
45 *	Caryophyllene oxide	19.86	12.95	–	–	–	–	–	–

* Identified by comparison with authentic samples. ^◊^ Identification by library data.

Extracts from plant species from the same family did not exhibit similar levels of repellency against *M. sjostedti* for all plant families evaluated. For example, the repellencies of the two *Cinnamomum* species of the Lauraceae family were similar and categorized as strong. However, the two species of *Satureja* from the Lamiaceae family showed different levels of repellency where Lemon savory (*S. biflora*) was classified as moderate and savory (*S. abyssinia*) as a weak repellent (Figure 2).

The extract from *P. nigrum* was the most repellent of the 24 plant extracts tested. On a related field study Oparaeke [7] observed that the application of 10% and 20% extracts of West African black pepper, *Piper guineense* (Schumacher), caused a significant reduction of *Megalurothrips* on flowers as compared to synthetic insecticide treatment and increased pod yield on cowpea. GC-MS analysis revealed β-caryophyllene, caryophyllene oxide and α-copaene as the major compounds in *P. nigrum* as also observed by Delétré *et al.* [36]. 

Apart from *P. nigrum*, plant extracts from the two *Cinnamomum* species also showed a strong repellent effect against *M. sjostedti*. Plant species of the genus *Cinnamomum* are fairly well known to have a repellent and toxic effect on several insect species such as the house fly *Musca domestica* [37], rice weevil *Sitophilus zeamais* [38], pulse beetle *Callasobruchus maculatus* [39] and mosquito, *Culex quinquefasciatus* [36,40,41]. Results of a chemical analysis were comparable to those found by Delétré *et al.* [28] who reported cinnamaldehyde at a similar quantity to be the major compound of *C. zeylanicum*.

Extracts from *C. myrrha*, which were classified as good repellents, had curzerene (75.27%) and β-elemene (13.52%) as major constituents. In previous studies, *Commiphora rostrata* Engler (Burseraceae) extracts showed repellency against the maize weevil [42], while extracts of *C. myrrha* and *C. holtziana* showed repellency against the poultry red mite, Dermanyssus gallinae (De Geer) (Mesostigmata: Dermanyssidae) [43]. The repellency of Lemongrass reported in the present study is also in line with a previous report on repellency activity of the essential oil of *C. citratus* against adults of *Culex quinquefasciatus* Say [44]. In terms of compositional analysis, our results were comparable to previous studies [36,45,46,47], where geranial and neral were identified as the major compounds in *C. citratus*.

Marjoram exhibited a good repellent effect against *M. sjostedti*. Similarly, van Tol *et al.* [13], reported *O. majorana* as a promising repellent against *Thrips tabaci* Lindeman (Thysanoptera: Thripidae). Moreover Yi *et al.* [48], observed potent fumigant toxicity of marjoram on the melon thrips, *Thrips palmi* Karny (Thysanoptera: Thripidae). In our study, Eucalyptus extract with 1–8 cineole as a major constituent was a good repellent against *M. sjostedti*. Oparaeke *et al.* [49], reported that the mean number of *M. sjostedti* was significantly reduced on plots sprayed with plant extracts mixed with *Eucalyptus* compared to unsprayed plots for two consecutive seasons. Similarly, Koschier and Sedy [50] reported repellency of 1,8-cineole (eucalyptol), a major constituent in rosemary oil, to female onion thrips. Citronella was also a good repellent against *M. sjostedti*. Similarly, Pinheiro *et al.* [51] reported that citronella grass, *C. winterianu*, showed enhanced insecticidal activity against the common blossom thrips, *Frankliniella schultzei* (Trybom) (Thysanoptera: Thripidae) and green peach aphid, *Myzus persicae* (Sulzer) (Hemiptera: Aphididae).

Among the eight moderate repellent extracts, Pennyroyal and Thyme exhibited the highest repellency against *M. sjostedti* at higher concentrations of 1%. Yi *et al.* [48], reported a 23.6-fold higher toxic effect than organophosphate dichlorvos against adult melon thrips. Essential oil from Thyme was reported to be highly repellent against western flower thrips *F. occidentalis* [20].

Eight extracts, *i.e.*, coriander, African blue basil, savory, solidage, ginger, conyza, lemon and rosemary were categorized as weak or non-repellent extracts. Similar results from Delétré *et al.* [36], showed that Lemon and Rosemary extracts did not exhibit a significant repellent effect against adults of *Anopheles gambiae* at a concentration of 1.0%, while Coriander, Ginger and Solidage showed slight repellent effect against *A. gambiae*. Nevertheless, Mayeku [52] reported that Conyza essential oil showed repellency and fumigant toxicity against *A. gambiae*. Rosemary was the least repellent among all the tested extracts. However, a higher concentration (10%) of Rosemary essential oil repelled female *T. tabaci* significantly in olfactometer bioassays [29]. Moreover, Rosemary essential oil at 0.1% and 1% concentrations decreased feeding damage of *T. tabaci* [53]. A possible reason for the varying results could be the low concentrations used in the present experiments as well as the composition of the Rosemary oil. For example, the major constituent in the Rosemary oil used in the olfactometer test by Koschier and Sedy [50] was 1,8-cineole (51%), whereas, the Rosemary extracts used in the current study contained <1% of 1,8-cineole. Another potential reason for the varying results could be the different behavioral responses between the two thrips species *M. sjostedti* and *T. tabaci*.

The chemical composition and broad spectrum of biological activity of essential oils, even from the same source, can be inherently variable for many reasons. Factors such as plant age, plant tissues used in the distillation process and type of distillation can cause variability of chemical composition of essential oils from a plant species [44]. Likewise, variability in behavioral and biological activity can be due to the age of the targeted pest organism [50,54,55]. Biological activity could be affected by interactions among structural components in the extracts. Even minor compounds can have a critical function due to coupled effects, additive action between chemical classes and synergy or antagonism [53].

The knowledge of extent, interaction and mode of inhibition of specific compounds in plant extracts may contribute to the successful application of pest management. Although several essential oils are repellent to thrips species in the laboratory, due to high volatility, the efficacy in the field is usually low [13,19,20]. Picard *et al.* [20] indicated that an application method incorporating the oils into polymeric mixture coatings to protect the bioactivity of the active compounds shows a better distribution and maintains high concentrations of active compounds on the surface of the leaves for a longer period. Many plant extracts are selective to certain pests, often biodegrade to nontoxic products and have few or no harmful effects on non target organisms and the environment [49,56,57]. They also can be useful to maximize thrips control efficiency and sustainability, while minimizing negative environmental effects. Integrating host plants with repellents (push) such as intercropping, row planting or border crop may improve the effectiveness of the formulated repellents in the field. However, all these applications need further study.

## 5. Conclusions

Our results provide evidence that female *M. sjostedti* are repelled by several plant extracts and that *P. nigrum*, *C. zeylanicum* and *C. cassia* are strong repellents. This indicates that plant extracts or phytochemicals have potential as natural pesticides for thrips control. The repellent effect could be related to the presence of different active compounds or a blend of odors, which induce an oriented movement away from the odor source. The biological activity of the major and most abundant compounds of the tested plant extracts should be further investigated under laboratory conditions to identify and evaluate specific behavioral responses of *M. sjostedti*. This will help to identify precisely the main modes of action and levels of bioactivity of different compounds to better integrate them into management strategies for legume flower thrips. Behavioral manipulation using natural products with fewer deleterious effects on non-targeted organisms and the environment for the management of thrips can be considered as a new approach for pest management in grain legumes.

## Supplementary Files

Supplementary File 1
