# Supplementary material for: Repellency of Plant Extracts against the Legume Flower Thrips Megalurothrips sjostedti (Thysanoptera: Thripidae)
Source: Insects. 2015 Jun 26;6(3):608–25. doi: 10.3390/insects6030608 (PMC4598655; doi:10.3390/insects6030608)
Supplement: Supplementary File 1 [file insects-06-00608-s001.zip › insects-78378-supplementary/insects-78378-supplement - final.pdf]

Supplement Materials

Table S1. Major compounds of least repellent plant extracts from different sources.

No	Common name	Botanical name	Major compounds	References
1	Thyme (common)	*T. vulgaris*	Thymol (35%), p-cymene (23%), carvacrol (15%)	[1]
2	Citronella	*Cymbopogon nardus*	Citronellal (35.5%), Geraniol (27.9%), Citronellol (10.7%)	[2]
3	Pennyroyal	*Mentha pulegium*	(+)-pulegone (87%)	IBMM, France
4	Satureja biflora	*Satureja biflora*	Linalool (50.60 %), germacrene D (10.63 %)	[3]
5	Geranium	*Pelargonium graveolens*	citronellol (41%)—geraniol (18%)	IBMM, France
6	Neem	*Melia azadirachta*	azadirachtin (,1%)	IBMM, France
7	Coriander	*Coriandrum sativum*	(+)-linalool (72%)	IBMM, France
8	Dill	*Anethum graveolens*	(+)-carvone (60%)—limonene (30%)	IBMM, France
9	African blue basil	*Ocimum kilimandscharicum*	Camphor (56.07%), DL-limonene (13.56%)	[4]
10	Ginger	*Zingiber officinale*	Zingiberene (30%)	IBMM, France
11	Litsea	*Litsea cubeba*	Geranial (45%), neral (32%)	IBMM, France
12	Thyme Borneol	*T hymus satureioides*	Borneol (31.2%), camphene (27.4%), α-pinene (17.5%) and linalool (6.3%)	[5]
13	Conza newii	*Conza newii*	(S)-(-)-perillyl alcohol, (S)-(-)-perillaldehyde, geraniol, (R)	[6]
14	Lemon	*Citrus limon*	Limonene (95%)	IBMM, France
15	Satureja abyssinica	*Satureja abyssinica*	pulegone (43.5%), isomenthone (40.7%)	[7]
16	Solidago	*Solidago canadensis*	Germacrene D (32%) - Limonene (13%)	IBMM, France
17	Rose mary	*Rosmalinus officinalis*	1,8-cineole (,1%), camphene (,1%), camphor (,1%)	IBMM, France

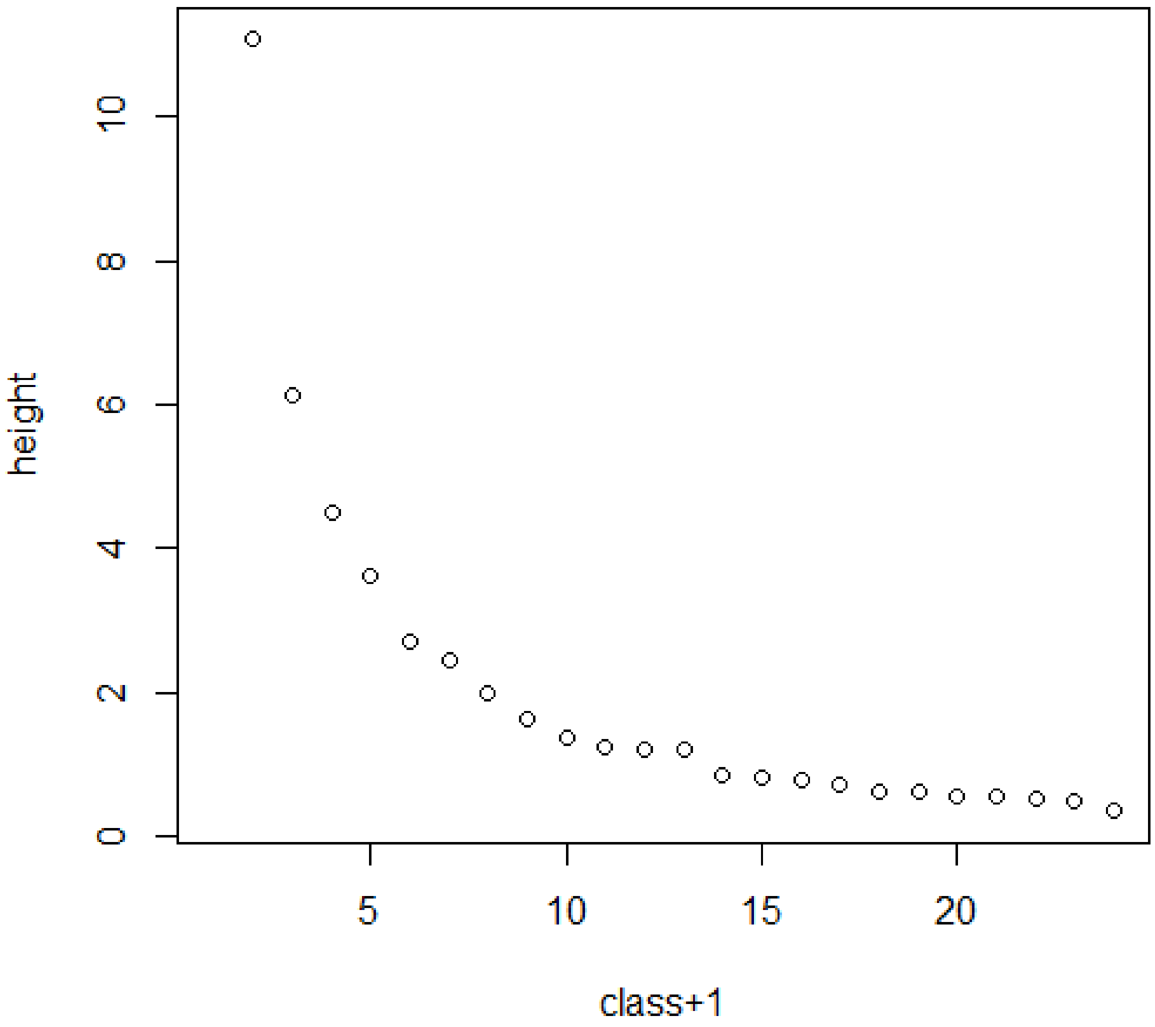

Figure S1. Branch height used to get the optimal number of classes in the tree which was determined by the decrease of the interclass variance.

References

1. IBMM—Institut des Biomolécules Max Mousseron, Montpellier, France. Avaliable online: http://www.ibmm.univ-montp1.fr/?lang=fr (accessed on 24 May 2015).

2. Koba, K.; Sanda, K.; Guyon, C.; Raynaud, C.; Chaumont, J.P.; Nicod, L. *In vitro* cytotoxic activity of *Cymbopogon citratus* L. and *Cymbopogon nardus* L. essential oils from Togo. *Bangladesh J. Pharmacol.* **2009**, *4*, 29–34.
3. Matasyoha, J.C.; Kiplimoa, J.J.; Karubiub, N.M.; Hailstorks, T.P. Chemical composition and antimicrobial activity of the essential oil of *Satureja biflora* (Lamiaceae) Bull. *Chem. Soc. Ethiop*. **2007**, *21*, 249–254.
4. Tantaoui-Elaraki, A.; Lattaoui, N.; Errift, A.; Benjilali, B. Composition and antimicrobial activity of essential oils of *Thymus broussonettii*. *J. Essent. Oil Res.* **1993**, *5*, 45–53.
5. Narwal, S.; Rana, A.C.; Tiwari, V.; Gangwani, S.; Sharma, R. Review on chemical constituents & pharmacological action of *Ocimum kilimandscharicum*. *Indo-Global J. Pharm. Sci.* **2011**, *4*, 287–293.
6. Mayeku, W.P.; Omollo, N.I.; Odalo, O.J.; Hassanali, A. Chemical composition and mosquito repellency of essential oil of *Conyza newii* propagated in different geographical locations of Kenya. *Med. Vet. Entomol.* **2013**, doi:10.1111/mve.12039.
7. Tolossa, K.; Asres, K.; El-Fiky, F.K.; Singab, A.N.B.; Bucar, F. Composition of the essential oils of *Satureja abyssinica* ssp. abyssinica and *Satureja paradoxa*: Their antimicrobial and radical scavenging activities. *J. Essential Oil Res.* **2007**, *19*, 295–300.
